# Effects of damage-regulated autophagy regulator gene on the SGC7901 human gastric cancer cell line

**DOI:** 10.3892/ol.2014.2220

**Published:** 2014-06-04

**Authors:** BAO-SONG ZHU, KUI ZHAO, XIN JIA, YONG-YOU WU, CHUN-GEN XING

**Affiliations:** Department of General Surgery, The Second Affiliated Hospital, Soochow University, Suzhou, Jiangsu 215021, P.R. China

**Keywords:** damage-regulated autophagy regulator, p53, autophagy, cell proliferation

## Abstract

The aim of this study was to investigate the effects of the adenoviral-mediated autophagy gene, damage-regulated autophagy regulator (DRAM), on the proliferation and autophagy of SGC7901 human gastric cancer cells *in vitro*. The recombinant adenovirus, AdMax-pDC315-DRAM-EGFP, working as a virus vector of DRAM was constructed and infected into the SGC7901 human gastric cancer cell line. The MTT assay was used to determine the growth rate of the SGC7901 cells. Activation of autophagy was monitored with monodansylcadaverin (MDC) staining following AdMax-pDC315-DRAM-EGFP treatment. Immunofluorescent staining was used to examine the expression of microtubule-associated protein 1 light chain 3 (LC3), and western blotting was used to examine the expression of apoptosis- and autophagy-associated proteins, including Beclin1, p53, p21 and B-cell lymphoma 2 (Bcl-2), in the culture supernatant. The viability of the SGC7901 cells was activated by AdMax-pDC315-DRAM-EGFP treatment. The AdMax-pDC315-DRAM-EGFP-treated cells exhibited positive LC3 expression detected by immunoreactivity and MDC staining. Inductions in the expression of the apoptosis-related proteins, p53 and p21, and the autophagic protein, Beclin1, were revealed by western blot analysis. By contrast, downregulation of the apoptosis-related protein, Bcl-2, following AdMax-pDC315-DRAM-EGFP treatment was identified. In conclusion, the present study demonstrated that AdMax-pDC315-DRAM-EGFP treatment resulted in upregulation of the level of autophagy and induction of cell proliferation in the SGC7901 human gastric cancer cell line *in vitro*.

## Introduction

Gastric cancer is the fourth most common type of cancer and the second leading cause of cancer-related mortality worldwide (1), with approximately one million new cases diagnosed each year. One of the major factors that controls tumor cell death is the tumor suppressor, p53 (2). The importance of cell death to tumor suppression is exemplified by p53 (3). In response to various forms of cellular stress, including DNA damage, hypoxia and oncogene activation, p53 levels are elevated (2). p53 has also been linked to another cell process that controls cell death known as autophagy (4,5). Autophagy is a vesicular trafficking process that mediates the degradation of long-lived proteins and is the only pathway within the cell for the degradation of organelles (6). In tumor development, autophagy is considered to act in either an oncogenic or tumor suppressive capacity and p53 has been reported to be an inducer of autophagy (4,5). Moreover, the discovery that damage-regulated autophagy regulator (DRAM), a p53 target gene which is required for p53-induced autophagy, is frequently downregulated in squamous cancers underscores the theory that autophagy is a component of tumor suppression downstream of p53 (5).

DRAM has been identified as an effector molecule that is critical for p53-mediated apoptosis, thus further supporting the tumor-suppressive role of autophagy (5,7,8). The discovery of DRAM revealed a novel role for autophagy in p53-induced apoptotic cell death (5), and DRAM is considered to be a crucial modulator in apoptosis and autophagy. The present study aimed to investigate the effects of AdMax-pDC315-DRAM-EGFP on growth, apoptosis and autophagy of gastric cancer cells *in vitro,* and to compare the infection efficiency, biological and molecular mechanisms of AdMax-pDC315-DRAM-EGFP.

## Materials and methods

### Reagents

The SGC7901 gastric cancer cell line was purchased from the Shanghai Institute of Cell Biology, Chinese Academy of Sciences (Shanghai, China). The RPMI-1640 medium was purchased from Gibco-BRL (Rockville, MD, USA). Fetal bovine serum (FBS) was obtained from Hangzhou Sijiqing Biological Engineering Material Co., Ltd. (Hangzhou, China), and L-glutamine and MTT were provided by Sigma (St. Louis, MO, USA). Antibodies against p53 (1:500; Rabbit monoclonal anti-human), B cell lymphoma 2 (Bcl 2; 1:500; Rabbit monoclonal anti-human), Beclin1 (1:700; Rabbit monoclonal anti-human) and p21 (1;500; Rabbit monoclonal anti-human) were supplied by Cell Signaling Technology, Inc. (Beverly, MA, USA).

### Adenoviral vectors and infections

The adenoviral vectors and NC-RNAi-GFP-AD were purchased from Shanghai Jikai Biological Technology Co., Ltd. (Shanghai, China). Stocks of replication-defective adenoviral vectors expressing green fluorescent protein (GFP) (AdMax-pDC315-DRAM-EGFP) were stored at −80°C. NC-RNAi-GFP-AD was used as a control which was also stored at −80°C. Infections were performed at 70–75% confluence in Dulbecco’s modified Eagle’s medium supplemented with 2% fetal calf serum (FCS). The cells were subsequently incubated at 37°C for at least 4 h, followed by the addition of fresh medium. Cells were then subjected to functional analyses at fixed time points following infection as described for individual experimental conditions (9).

### Determination of optimal multiplicity of infection (MOI)

The SGC7901 cells (1×10^4^ cells/well) were seeded in 96-well plates and reached 60–70% confluence. Different MOI (MOI = 10, 20, 30, 50 and 100) values of the NC-RNAi-GFP-AD 100-μl diluted infected cells were added to the plates and, after 8 h, RPMI-1640 medium containing 10% FBS was added. After 48 h of culture, the cells were counted under a fluorescence microscope (Leica DMI4000B; Leica Microsystems Wetzlar GmbH, Wetzlar, Germany) to calculate the number of cells expressing GFP.

### Cell culture and viability assay

The SGC7901 cells were maintained in RPMI-1640 medium containing 10% heat-inactivated FBS and 0.03% L-glutamine, and incubated in an atmosphere of 5% CO_2_ at 37°C. The cells in a mid-log phase were used in the experiments. Cell viability was assessed by the MTT assay. To determine the effects of AdMax-pDC315-DRAM-EGFP, the SGC7901 cells were plated into 96-well microplates (7×10^4^ cells/well) and AdMax-pDC315-DRAM-EGFP was added to the culture medium. Cell viability was assessed by the MTT assay 24 h after AdMax-pDC315-DRAM-EGFP treatment. MTT (Sigma) solution was added to the culture medium (500 μg/ml final concentration) for 4 h prior to the end of treatment and the reaction was inhibited by the addition of 10% acid sodium dodecyl sulfate (100 μl; Beijing Biosea Biotechnology Co., Ltd., Beijing, China). The absorbance value (A) at 570 nm was measured using an automatic multi-well spectrophotometer (Bio-Rad, Richmond, CA, USA). The percentage of cell proliferation was calculated as follows: Cell proliferation (%)= (1−A of experiment well/A of positive control well) × 100.

### Visualization of MDC-labeled vacuoles

Exponentially growing cells were plated on 24-chamber culture slides, cultured for 24 h and then incubated with the drug in 10% FCS/RPMI-1640 medium for 12 and 24 h. Autophagic vacuoles were labeled with MDC (Sigma) (10) by incubating cells with 0.001 mmol/l MDC in RPMI-1640 at 37°C for 10 min. Following incubation, cells were washed three times with phosphate-buffered saline (PBS) and immediately analyzed with a fluorescence Nikon Eclipse TE300 microscope (Nikon, Tokyo, Japan) equipped with a filter system (V-2A excitation filter, 380–420 nm; barrier filter, 450 nm). Images were captured with a charged couple device camera (CoolSNAP ES, Roper Scientific; Trenton, NJ, USA) and imported into Photoshop.

### Immunofluorescent staining

The SGC7901 cells were seeded onto 24-chamber culture slides and treated with AdMax-pDC315-DRAM-EGFP. Following fixation in methanol for 10 min, cells were blocked with a buffer containing 1% bovine serum albumin (BSA; Hangzhou Sijiqing Biological Engineering Material Co., Ltd.) and 0.1% Triton X-100 (Nanjing KeyGen Biotech., Co., Ltd., Nanjing, China) for 1 h. The cells were then incubated with the primary antibody against LC3 (diluted 1:200; Santa Cruz Biotechnology, Inc., Santa Cruz, CA, USA) and PBS containing 1% BSA at 4°C overnight, and then incubated for 1 h with secondary ghost against rabbit cy3 fluorescence conjugated antibodies (1:500; Sigma) to visualize the binding sites of the primary antibody with laser confocal microscopy (Leica Microsystems Wetzlar GmbH).

### Total cell protein extraction and western blot analysis

For extraction of total cell proteins, cells were washed with pre-cooled PBS and subsequently lysed in pre-cooled radioimmunoprecipitation assay lysis buffer [50 mM Tris-HCl (pH 7.4), 150 mM NaCl, 1 mM dithiothreitol, 0.25% sodium deoxycholate and 0.1% NP-40] containing 1 mM phenylmethysulfonyl fluoride, 50 mM sodium pyrophosphate, 1 mM Na_3_VO_4_, 1 mM NaF, 5 mM EDTA, 5 mM EGTA and protease inhibitors cocktail (Nantong Biyuntian Biological Technology Co., Ltd., Nantong, China). Cell lysis was performed on ice for 30 min. Clear protein extracts were obtained by centrifugation 12,000 × g for 30 min at 4°C. Protein extraction from the SGC7901 gastric cancer cells was performed as previously described (11). Protein concentration was determined with a Bradford protein assay kit (Nanjing KeyGen Biotech., Co., Ltd.). Proteins were resolved on 8.5% polyacrylamide gels (Nantong Biyuntian Biological Technology Co., Ltd.) and subsequently transferred onto nitrocellulose membranes (Nanjing KeyGen Biotech., Co., Ltd.). For immunoblotting, nitrocellulose membranes were incubated with specific antibodies recognizing target proteins overnight at 4°C. The membranes were washed as previously described and then incubated with horseradish peroxidase-conjugated goat anti-rabbit IgG monoclonal secondary antibody (1:20,000; Amersham Pharmacia Biotech, Arlington Heights, IL, USA) for 1 h at room temperature and and visualized by autoradiograpy. β-actin protein (1:5,000; Sigma) was used as the loading control. The membrane was washed three times with Tris-buffered saline and Tween 20 [10 mM Tris-HCl (pH 8.0), 150 mM NaCl and 0.5% Tween-20] and developed using the enhanced chemiluminescence detection system (Amersham Pharmacia Biotech). The intensity of the immunoreactive bands was quantified using a densitometer (SI, Molecular Dynamics, Sunnyvale, CA, USA).

### Statistical analysis

All data are presented as the mean ± standard deviation. Statistical analysis was performed by analysis of variance followed by Dunnett’s test. P<0.05 was considered to indicate a statistically significant difference.

## Results

### AdMax-pDC315-DRAM-EGFP treatment increases cell viability

**Figure 1:**
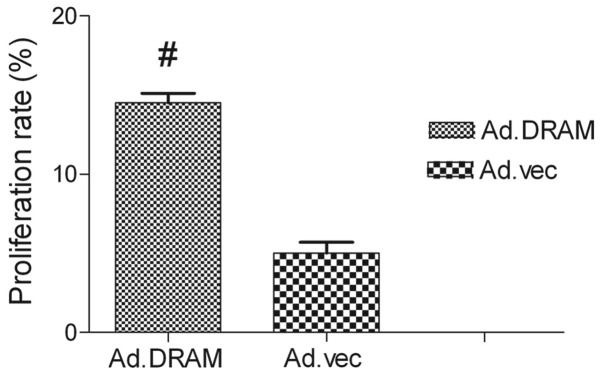
Reduced cell viability following AdMax-pDC315-DRAM-EGFP treatment. SGC7901 cells (7×10^4^ cells/ml) were cultured with AdMax-pDC315-DRAM-EGFP (MOI, 60) for 24 h and cell viability was analyzed by the MTT assay. Values are expressed as the mean ± standard deviation of three independent experiments. ^#^P<0.05, compared with the control group. DRAM, damage-regulated autophagy regulator; MOI, multiplicity of infection; vec, vector.

The MTT assay showed that the proliferation capacity of gastric cancer cells infected with AdMax-pDC315-DRAM-EGFP was significant1y higher than AdMax-pDC315-EGFP (MOI, 60) (P<0.05). The DRAM gene promoted the proliferation of cell viability. After 24 h of treatment, the rate of proliferation had reached 14.71±4.13% (Fig. 1).

### Infection efficiency and cell morphology

**Figure 2:**
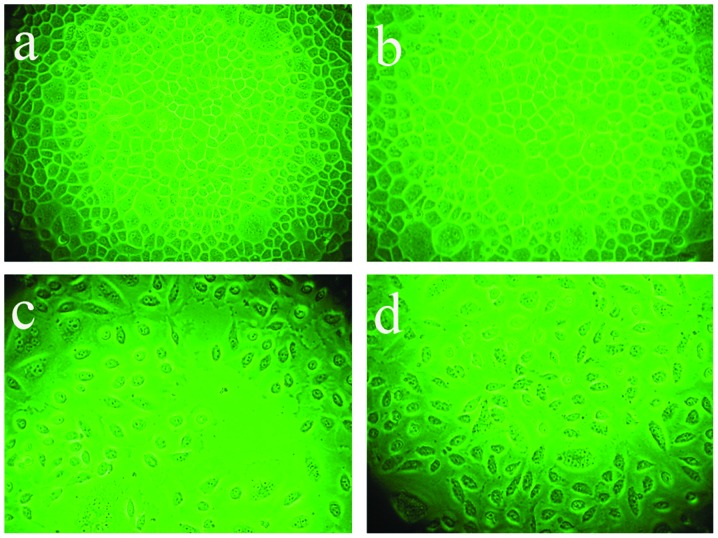
Infection efficiency and cell morphology were analyzed under a fluorescence microscope following AdMax-pDC315-DRAM-EGFP (MOI, 60) and AdMax-pDC315-EGFP (MOI, 60) treatment. The SGC7901 cells were incubated with AdMax-pDC315-DRAM-EGFP (MOI, 60) for the indicated time. (A) Control, (B) AdMax-pDC315-EGFP, (C) 12 h after AdMax-pDC315-DRAM-EGFP (MOI, 60) treatment and (D) 24 h after AdMax-pDC315-DRAM-EGFP (MOI, 60) treatment. Magnification, ×200. DRAM, damage-regulated autophagy regulator; MOI, multiplicity of infection.

Following AdMax-pDC315-DRAM-EGFP (MOI, 60) infection (12 h) of SGC7901 cells, the cell body appeared swollen, rounded and the cells revealed deformation, with the cells showing further deformation after 24 h. After infection for 24 h, the SGC7901 cells were counted under a fluorescence microscope to determine the percentage of the of infected cells (Fig. 2). Infection efficiency did not increase with increasing MOI and the time of infection. The infection efficiency and cell viability are dependent on the correct MOI (60) and the time of infection (24 h). It was determined that at an MOI of 60, the infection efficiency was 93±5.4%.

### AdMax-pDC315-DRAM-EGFP infection increases autophagic vacuoles

**Figure 3:**
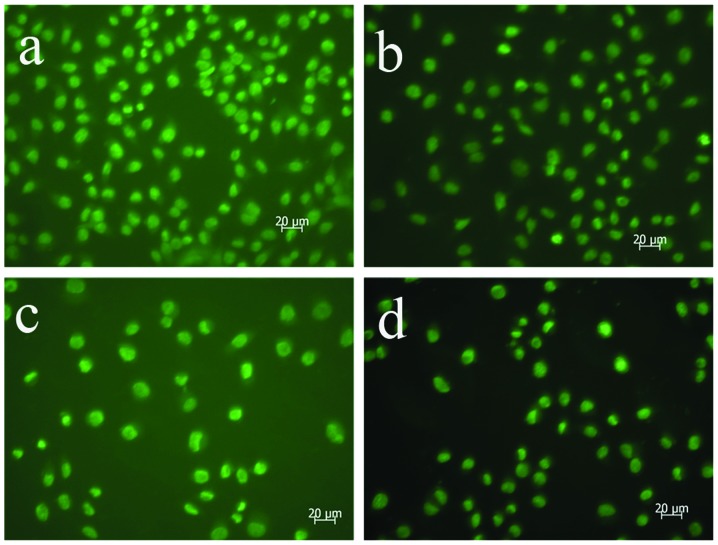
MDC staining revealed autophagy was activated following AdMax-pDC315-DRAM-EGFP (MOI, 60) and AdMax-pDC315-EGFP (MOI, 60) treatment. The SGC7901 cells were incubated with AdMax-pDC315-DRAM-EGFP (MOI, 60) for an indicated time and stained with MDC (100 μmol/l). Fluorescent particles revealed late autophagic vacuoles. (A) Control, (B) AdMax-pDC315-EGFP, (C) 12 h after AdMax-pDC315-DRAM-EGFP (MOI, 60) treatment and (D) 24 h after AdMax-pDC315-DRAM-EGFP (MOI, 60) treatment. Magnification, ×1,000. MDC, monodansylcadaverin; MOI, multiplicity of infection; DRAM, damage-regulated autophagy regulator.

The autofluorescent substance, MDC, is a marker for late autophagic vacuoles (L-AVs), but not endosomes (12). The dye is trapped in acidic, membrane-rich organelles and exhibits an increased fluorescence quantum yield in response to the compacted lipid bilayers present in L-AVS (10). When cells are analyzed under a fluorescent microscope, AVs stained by MDC appear as distinct dot-like structures distributed within the cytoplasm or localizing in the perinuclear regions. In this study, an increase in the number of MDC-labeled vesicles following infection with AdMax-pDC315-DRAM-EGFP (MOI, 60) from 12 to 24 h was observed (Fig. 3).

### AdMax-pDC315-DRAM-EGFP infection upregulates the expression of LC3

**Figure 4:**
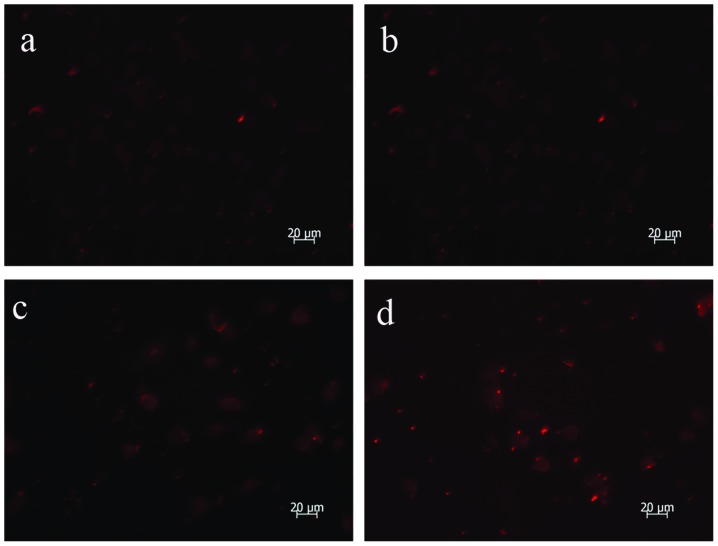
Microtubule-associated protein 1 light chain 3 expression and location in SGC7901 cells following treatment with AdMax-pDC315-DRAM-EGFP. (A) Control and (B) AdMax-pDC315-EGFP control (magnification, ×1,000) (n=3). Cells were treated with AdMax-pDC315-DRAM-EGFP (multiplicity of infection, 60) for (C) 12 and (D) 24 h, and observed by immunofluorescence microscopy. AdMax-pDC315-DRAM-EGFP increased the punctuate distribution of LC3 from 12 to 24 h. DRAM, damage-regulated autophagy regulator.

Microtubule-associated protein 1 LD3, the mammalian ontology of Atg8, targets to the autophagosomal membranes in an Atg5-dependent manner and remains there even after Atg12-Atg5 dissociates. LC3 is considered to be the only credible marker of the autophagosome in mammalian cells (13). The present study used immunofluorescence to analyze the expression and location of LC3 and identified an increased formation of autophagosomes following AdMax-pDC315-DRAM-EGFP (MOI, 60) infection (Fig. 4).

### AdMax-pDC315-DRAM-EGFP infection upregulates the expression of Beclin1 and p53

**Figure 5:**
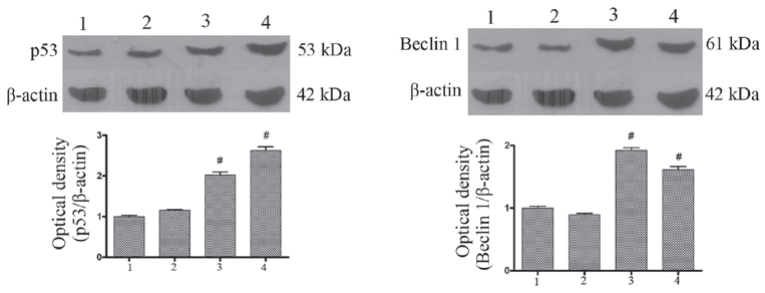
Effect of AdMax-pDC315-DRAM-EGFP (MOI, 60) infection on p53 and Beclin1 protein expression. The SGC7901 cells were treated with AdMax-pDC315-DRAM-EGFP (MOI, 60) and AdMax-pDC315-EGFP (MOI, 60) for 12 and 24 h, then harvested for extraction of total proteins. AdMax-pDC315-DRAM-EGFP upregulated the expression of p53 and Beclin1 protein. 1, normal group; 2, AdMax-pDC315-EGFP group; 3, AdMax-pDC315-DRAM-EGFP treatment for 12 h; 4, AdMax-pDC315-DRAM-EGFP treatment for 24 h. Statistical comparisons were performed using Dunnett’s test (n=3). Values are expressed as the mean ± standard deviation. ^#^P<0.01, compared with the control group. Cont, control; DRAM, damage-regulated autophagy regulator; MOI, multiplicity of infection.

To investigate the effects of AdMax-pDC315-DRAM-EGFP (MOI, 60) infection on the expression of autophagic-related proteins, western blot analysis was used to detect the expression of p53 and Beclin1. The findings revealed that the basal level of Beclin1 and p53 in the SGC7901 cells was low. Following incubation with AdMax-pDC315-DRAM-EGFP (MOI, 60), the Beclin1 and p53 protein expression levels significantly increased from 12 to 24 h (Fig. 5).

### AdMax-pDC315-DRAM-EGFP infection increases the expression of p21 and decreases the expression of Bcl-2

**Figure 6:**
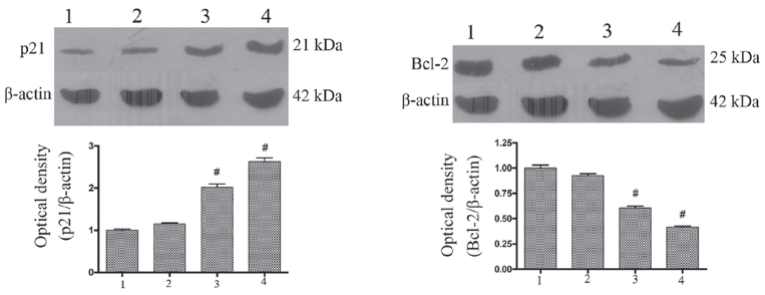
Effects of AdMax-pDC315-DRAM-EGFP infection on Bcl-2 and p21 protein expression in SGC7901 cells. (A) Effects of AdMax-pDC315-DRAMEGFP (MOI, 60) on Bcl-2 and p21 protein expression. The SGC7901 cells were treated with AdMax-pDC315-DRAM-EGFP (MOI, 60) and AdMax-pDC315-EGFP (MOI, 60) for 12 or 24 h then harvested for extraction of total proteins. AdMax-pDC315-DRAM-EGFP upregulated the expression of p21 and downregulated the expression of Bcl-2 protein. 1, normal group; 2, AdMax-pDC315-EGFP group; 3, AdMax-pDC315-DRAM-EGFP treatment for 12 h; 4, AdMax-pDC315-DRAM-EGFP treatment for 24 h. Statistical comparisons were performed using Dunnett’s test (n=3). Values are expressed as the mean ± standard deviation. ^#^P<0.01, compared with the control group. Cont, control; DRAM, damage-regulated autophagy regulator; Bcl-2, B-cell lymphoma 2; MOI, multiplicity of infection.

To determine whether AdMax-pDC315-DRAM-EGFP (MOI, 60) infection affects the expression of apoptotic-related proteins, western blot analysis was used to detect the expression of Bcl-2 and p21 (Fig. 6). The findings revealed that the basal level of p21 protein in SGC7901 cells was low; however, following incubation with DRAM, the p21 protein expression levels were significantly increased from 12 to 24 h. By contrast, the Bcl-2 protein expression levels were downregulated with the addition of AdMax-pDC315-DRAM-EGFP (MOI, 60) (Fig. 6).

## Discussion

A number of studies have found that the baseline levels of autophagy act as a tumor suppressor mechanism. Nevertheless, stress-induced autophagy constitutes a major pro-survival mechanism for tumors exposed to a hypoxic microenvironment or to chemotherapeutic agents. Thus, autophagy mediates either antitumor or pro-tumor functions (14,15), and hence, is considered as a ‘double-edged sword’ in oncogenesis and tumor progression (16).

The genetic inactivation of p53, the best-known human oncosuppressor protein, has been observed in >50% of all types of human cancer and mostly mediates tumor suppression, not only by transactivating pro-apoptotic and cell cycle arresting genes, but also by regulating autophagy. p53 mutations that simultaneously abolish its pro-apoptotic and autophagy-inhibitory functions behave as ‘multi-hit’ events, as opposed to ‘single-hit’ mutations that only affect the classical (pro-apoptotic and/or cell cycle-arresting) functions of the p53 system (17,18).

Under genotoxic stress, p53 has been shown to upregulate the transcription of DRAM. DRAM, a 238-amino acid protein, which is highly conserved in higher eukaryotes, is localized to the lysosomal membrane. Knockdown of DRAM expression promoted survival following exposure to DNA-damage, and DRAM is also required for p53-induced autophagy and cell death (5).

In the present study, the autophagic level is low in the SGC7901 gastric cancer cell line; however, with the addition of DRAM adenovirus the autophagy-specific marker, LC3, was upregulated indicating an increased formation of autophagosomes induced by DRAM infection. Beclin1, the mammalian ortholog of the yeast apg6/vps30 gene, plays a role in two fundamentally important cell biological pathways, autophagy and apoptosis. Beclin1 is a major determinant in the initiation of autophagy (18–21).

Beclin1 is monoallelically deleted in human breast and ovarian cancers and is expressed at reduced levels in those tumors (22,23). The findings of the present study suggest that autophagy is induced by DRAM and its activation may not contribute to the antitumor effects of DRAM. Moreover, DRAM increased the expression of Beclin1, particularly the production of p53. Bcl-2 and Bcl-xL are associated with the evolutionarily conserved autophagy inducer, Beclin1, a haplo-insufficient tumor suppressor (24), and inhibit autophagy (25). The inhibition may require Bcl-2 to localize on the endoplasmic reticulum (25,26) and, notably, the BH3 domain of Beclin1 mediates their association (27). In the present study, the expression of Beclin1 was upregulated with the treatment of DRAM and the expression of Bcl-2 was decreased, indicating that DRAM may have decreased the expression of Bcl-2. The cell-cycle-regulating protein, p21, is a cyclin-dependent kinase inhibitor coupled to a wide variety of cell functions, including p53-dependent growth suppression, cell cycle arrest following DNA damage, and the inhibition and induction of apoptosis (28). In our study, following DRAM infection, the expression of p53 was upregulated simultaneously to the increase of p21.

In conclusion, the level of autophagy increased with the addition of DRAM, which also induced proliferation of the SGC7901 cells. The integrative effect of autophagy induced by DRAM may have activated the proliferation of the SGC7901 cells. These findings suggest that autophagy induces the survival of the SGC7901 tumor cell line. Further study should investigate the effects of DRAM on primary culture gastric cancer cells collected from patients with gastric cancer. This may provide novel treatment strategies for patients with gastric cancer.
